# Non-linear frequency doubling up-conversion of geothermal radiation cannot provide sufficient light to support oxygenic photosynthesis at deep-sea hydrothermal vents

**DOI:** 10.1093/nsr/nwaf291

**Published:** 2025-07-18

**Authors:** Conrad W Mullineaux, Christopher D P Duffy

**Affiliations:** School of Biological & Behavioural Sciences, Queen Mary University of London, UK; School of Biological & Behavioural Sciences, Queen Mary University of London, UK; Digital Environment Research Institute, Queen Mary University of London, UK

Photosynthesis on planet Earth is generally assumed to require sunlight. However, a green sulfur bacterium was shown to grow photosynthetically in regions of the ocean too deep for sunlight to penetrate, by exploiting the infra-red geothermal radiation from deep-sea hydrothermal vents [1]. The total emitted photon flux (radiance) from these vents across the whole 600–1000 nm spectral window is estimated at 10^11^ photons cm^−2^ s^−1^ sr^−1^, or 10^8^ photons cm^−^^2^ s^−1^ sr^−1^ at 750 ± 50 nm [1]. Such radiance is thought to be sufficient to allow extremely slow growth of this anoxygenic phototroph very close to the black smoker chimneys, aided by its huge chlorosome light-harvesting antenna with peak absorbance at about 750 nm [1].

Cyanobacterial genomes can be identified in metagenomic samples from deep-sea hydrothermal vents [2]. The oxygenic photosynthesis of cyanobacteria is much more challenging to sustain by geothermal radiation than the anoxygenic photosynthesis of green sulfur bacteria. Oxygenic photosynthesis requires shorter (400–750 nm)-wavelength photons carrying sufficient energy to drive water-splitting at Photosystem II. The red limit for oxygenic photosynthesis was traditionally thought to be at 700 nm, but some cyanobacteria have been shown to exploit light at slightly longer wavelengths for limited growth and survival in environments deficient in visible light, by using specialized red-shifted variants of chlorophyll [3]. Oxygenic photosynthesis also requires a higher flux of absorbed photons, because eight photons in total are needed to release one molecule of O_2_ and pass the electrons through Photosystem II and then Photosystem I, and several steps are multi-electron/photon processes involving intermediates that are only meta-stable on 1–10 ms timescales. Nevertheless, growth by oxygenic photosynthesis appears to be sustainable at least down to incident fluxes (irradiances) of 2.4 × 10^12^ photons cm^−2^ s^−1^, as observed for algae growing beneath the arctic ice pack [4]. The problem is that geothermal radiation from hydrothermal vents is insufficient in the wavelength range required by cyanobacteria.

Li *et al*. recently suggested that this problem could be resolved by invoking frequency doubling up-conversion [2]. Up-conversion is an optical phenomenon in which two photons interact simultaneously with a non-linear material to generate a single photon with twice the energy and half the wavelength. A similar phenomenon is becoming familiar to cell biologists through its exploitation in two-photon fluorescence microscopy, in which fluorophores absorbing in the visible range are excited by a combination of two photons from an infra-red laser [5]. In both cases, the phenomenon requires extremely high photon fluences in order to give two photons a reasonable chance of simultaneous interaction. This is typically achieved by compressing the photon flux from a laser into ultrashort pulses [usually in the femtosecond (fs) range] and focusing the laser onto a tiny spot. Li *et al.* demonstrated up-conversion in chalcopyrite and other minerals found in hydrothermal vents using a highly focused pulsed laser [2]. The efficiencies achieved are low (10^−14^, up to 10^−7^ at high pressure), but at realistic hydrothermal vent radiances they must be far lower. Taking the conditions reported for the experiment in their Fig. 1d, with a 1.5 mW, 928 nm laser with photons compressed into 130 fs pulses at 76 MHz frequency and focused into a 10 µm^2^ spot [2], we can crudely estimate a photon flux density within each pulse of about 7 × 10^27^ photons cm^−2^ s^−1^ (for comparison, full sunlight is about 60 billion times weaker at 1.2 × 10^17^ photons cm^−2^ s^−1^). The efficiency of up-conversion is proportional to light intensity (Fig. 1d from [2]), so it is straightforward to extrapolate down to a more realistic hydrothermal vent radiance. Taking the reasonable estimate of 10^12^ photons cm^−2^ s^−1^ sr^−1^ suggested by Li *et al.* [2], that equates to an irradiance of about 10^13^ photons cm^−2^ s^−1^ in the best possible case where all photons in all directions are collected. The efficiency of up-conversion will then be around 10^−29^ (Fig. 1d from Li *et al*. [2]), and the frequency-doubled emission will be around 10^−16^ photons cm^−2^ s^−1^. The high-pressure effect (Fig. 1f from Li *et al.* [2]) might increase the efficiency by a factor of 10^7^, giving 10^−^^9^ photons cm^−2^ s^−^^1^. That's still only 1 photon per cm^2^ every 30 years and is about 2 billion trillion times lower than the lowest irradiance ever observed to support oxygenic photosynthesis [4]. In summary, the up-conversion observed here appears irrelevant to potential oxygenic photosynthesis at hydrothermal vents.

Maybe there is some other mechanism that could generate sufficient photons in the visible range to support oxygenic photosynthesis, but such bright light has never been observed at hydrothermal vents. Li *et al*. [2] quote an observation of 400–600 nm light at 10^4^ photons cm^−2^ s^−^^1^ sr^−1^ [6]. It's about 10 times more than expected from black-body radiation [6] (and about 100 trillion times more than expected from up-conversion), but it's still over 20 million times too weak to support oxygenic photosynthesis. Sufficiently bright visible light would be very conspicuous in the ocean depths. It seems more plausible to look for other explanations for the cyanobacterial genomes found at hydrothermal vents. Li *et al.* make a good argument that the vent cyanobacteria appear to be a specialized population rather than just debris settling down from the photic zone [2], but cyanobacteria can be surprisingly versatile in their metabolism and habitats. Very relevant to the current discussion, cyanobacteria were found growing in total darkness in deep subsurface rock samples from southern Spain [7]. Just like the vent cyanobacteria discussed by Li *et al.* [2], the Spanish subsurface cyanobacteria retain a full set of photosynthesis genes (including all components of both photosystems, cytochrome *b*_6_*f*, the phycobilisomes and the chlorophyll biosynthesis pathway) [7], but they are clearly not growing photosynthetically. Puente-Sánchez *et al.* argue that the cyanobacteria grow chemolithoautotrophically with hydrogen as their electron donor [7]. Something similar could surely be happening at hydrothermal vents, which are often copious emitters of hydrogen [8].
